# Enhancement of Mammographic Images Using Histogram-Based Techniques for Their Classification Using CNN

**DOI:** 10.3390/s23010235

**Published:** 2022-12-26

**Authors:** Khalaf Alshamrani, Hassan A. Alshamrani, Fawaz F. Alqahtani, Bander S. Almutairi

**Affiliations:** 1 PhD Radiological Sciences Department, College of Applied Medical Sciences, Najran University, Najran 6641, Saudi Arabia; 2 Radiology Department, King Abdulaziz University Hospital, Jeddah 3646, Saudi Arabia

**Keywords:** malignant calcifications, cancer classification, HIW, CLAHE, mammography histogram, CAD system

## Abstract

In the world, one in eight women will develop breast cancer. Men can also develop it, but less frequently. This condition starts with uncontrolled cell division brought on by a change in the genes that regulate cell division and growth, which leads to the development of a nodule or tumour. These tumours can be either benign, which poses no health risk, or malignant, also known as cancerous, which puts patients’ lives in jeopardy and has the potential to spread. The most common way to diagnose this problem is via mammograms. This kind of examination enables the detection of abnormalities in breast tissue, such as masses and microcalcifications, which are thought to be indicators of the presence of disease. This study aims to determine how histogram-based image enhancement methods affect the classification of mammograms into five groups: benign calcifications, benign masses, malignant calcifications, malignant masses, and healthy tissue, as determined by a CAD system of automatic mammography classification using convolutional neural networks. Both Contrast-limited Adaptive Histogram Equalization (CAHE) and Histogram Intensity Windowing (HIW) will be used (CLAHE). By improving the contrast between the image’s background, fibrous tissue, dense tissue, and sick tissue, which includes microcalcifications and masses, the mammography histogram is modified using these procedures. In order to help neural networks, learn, the contrast has been increased to make it easier to distinguish between various types of tissue. The proportion of correctly classified images could rise with this technique. Using Deep Convolutional Neural Networks, a model was developed that allows classifying different types of lesions. The model achieved an accuracy of 62%, based on mini-MIAS data. The final goal of the project is the creation of an update algorithm that will be incorporated into the CAD system and will enhance the automatic identification and categorization of microcalcifications and masses. As a result, it would be possible to increase the possibility of early disease identification, which is important because early discovery increases the likelihood of a cure to almost 100%.

## 1. Introduction

Breast cancer is now the second leading cause of female mortality worldwide, after coronary heart disease. The malignant transformation of mammary gland cells causes this disease. In healthy organisms, cells grow and divide slowly, allowing new cells to replace old or damaged ones. When the genes that control these processes become mutated, the cells divide uncontrollably, giving rise to a nodule or tumor. Breast cancer is staged based on tumor size, lymph node involvement, and other organ or metastasis involvement [1].

Mammography detects breast lesions up to two years before they become palpable, have invaded other tissues in depth, or have spread to lymph nodes and other organs. Mammography detects 90% of tumors, while physical examination detects less than 50%. Finally, blood and urine tests can detect cancer, but it is rare [2]. A mammography is a low-dose X-ray examination that yields pictures of the breast. Due to its ability to often identify changes in the breast before a patient or doctor can feel them, mammography is crucial in the early identification of breast cancer. Annual mammograms may aid in the early diagnosis of breast cancers, when they are most treatable and breast-conservation medicines are accessible, according to research. Ductal carcinoma in situ (DCIS), abnormal cells in the lining of a breast duct that may progress to invasive cancer, may also be discovered by mammography. The only breast cancer screening method that has been shown to lower the number of fatalities is mammography; it is possible for the network to learn a set of characteristics that allows obtaining a classification of new input data. Mammograms are X-ray images of the mammary gland. The two views are the cranial-caudal (CC) and the mediolateral-oblique (MLO) [2,3]. The CC view clearly shows fat, lateral glandular tissue, and the pectoral muscle at the image margin. One of the major flaws of mammography-related CAD systems is their inability to distinguish image content. It will increase the percentage of correctly classified images in one of those subsets during pre-classification. Increasing the contrast between different structures is one type of enhancement algorithm. The goal is to make the pathological and healthy breast tissue more distinct. This paper implements two histogram-based contrast algorithms: HIW and CLAH (CLAHE) [4]. Different parameters of both algorithms were tested throughout the work to see which produced the best results [5]. A unique type of adaptive histogram equalization is called CLAHE. By adaptively boosting each pixel’s contrast to its immediate surroundings, adaptive histogram equalization increases the contrast over the whole picture. All contrast levels (small and large) in the original picture are enhanced by this method. Local histograms are created by calculating histograms for discrete geographic regions of pixels to improve local contrast using adaptive histogram equalization.

Intensity windowing has a variation known as HIW. A specific sub range of the picture intensity values may be given the full contrast of the display device thanks to intensity windowing. The entire image is turned black (for values below the intensity window range’s lowest value) or white for any areas with values outside the chosen intensity window range (for values above the maximum value of the intensity window range). HIW customizes standard intensity windowing by choosing the intensity window range for each image (background, labels) by statistically analyzing each image’s histogram, finding the “humps” or modes of the histogram, and determining which modes represent the various breast tissue types (fatty, dense, muscle), or other parts of the image.

There are different techniques used in the frequency domain, including discrete co-sine transform (DCT), dual tree-complex wavelet (DT-CW), discrete wavelet transform (DWT), fast Fourier transform (FFT), and lifting wavelet transform (LWT). Nonetheless, these methods may cause blurriness in the resulting output image and noise emphasis towards the edges.

A CAD system based on convolutional neural networks (CNN) [6] was used to evaluate these algorithms. This network divides mammograms into three categories: benign, malignant, and healthy tissue. Enhancement algorithms are used before classification to improve classification accuracy. Thus, this work aims to improve image quality to help a detection system find and analyze breast lesions and aid CAD using convolutional neural networks. In this way, it hopes to help fight against breast cancer by assisting and supporting physicians. These methods are not intended to modify or increase the information present on the mammogram, but simply to enhance contrast by enhancing the intrinsic features of the mammogram. The second-largest cause of death among women today, behind coronary heart disease, is breast cancer. This illness is brought on by the malignant alteration of mammary gland cells.

To assist CAD in employing convolutional neural networks and to help a detection system discover and analyze breast lesions, this effort intends to increase picture quality. Pre-processing mammographic pictures using histogram-based contrast enhancement algorithms may greatly increase a CAD system’s pre-processing step. Breast tissue anomalies were identified and categorized using convolutional neural networks. CLAHE is a variant of adaptive histogram equalization (AHE), which takes care of over-amplification of the contrast. CLAHE operates on small regions in the image, called tiles, rather than the entire image. If the existing technology of projecting mammograms on film is not replaced with a softcopy display system, such customization will not be readily accomplished. The effectiveness of digital mammography will ultimately rely on increased diagnostic accuracy compared to that of traditional screen-film mammography, given the additional expenditures. The acceptability of this new technology will be significantly influenced by the development and evaluation of image-processing techniques that enable the identification and characterization of certain lesion types.

## 2. Literature Review

Table 1 shows the Results of comparing the study with some of the other studies that aimed to improve breast cancer diagnostic images using several techniques. They were compared in terms of samples, methods, results, and the contribution of each study [7,8,9,10,11,12,13,14,15,16,17,18].

The prognosis for cancer predicts the patient’s outcome. A prompt and precise diagnosis and prognosis will be very beneficial for cancer patients. DL has become a popular technique as a result of the availability of powerful computer resources. Most of a typical computer-aided design (CAD) system is made up of things such as preprocessing, feature identification, feature extraction and selection, classification, and performance evaluation. Such a framework may be modeled using the deep learning (DL) method, and the model is made robust by using multilayer, fully connected neural nets. The construction of an artificial neural network (ANN) classifier based on statistical learning of machining vibrations is shown. In light of turning procedures integrating feature calculation, choice, and classification, six unique tool flaws have been examined. With an accuracy of 93.33%, the output of the trained ANN is used to classify the fault and fault-free conditions in the cutting tool. The effectiveness of this model has since been evaluated in comparison to machine learning (ML) classifiers. Since the deep learning-based ANN model demonstrates greater accuracy, it may be recommended for use in condition monitoring of single-point cutting tools [19].

## 3. Materials and Methods

### 3.1. Dataset Description

The images used in this work come from the DDSM database [20]. Depending on the information it contains, highlighting the assessment of the mammogram and the type of anomalies, five types of mammograms can be differentiated:

- Images with benign microcalcifications
- Images with malignant microcalcifications.
- Images with benign masses.
- Images with malignant masses.
- Images with healthy tissue.

The mammographic image analysis research community may use the Digital Database for Screening Mammography (DDSM) as a resource. The database’s main goal is to assist reliable research in the creation of computer algorithms that will help with screening. The database could also be used to make tools for teaching or training, as well as algorithms for diagnosing problems.

Due to the high number of mammograms contained in the DDSM, it is not feasible to work with the complete database, since the training and validation time of the system would be very long. For this reason, a set of mammograms (a training set) has been selected that allows the evaluation of the results of the work in an acceptable timeframe. The training set used in the work has ten mammograms from each set of images. Of the ten mammograms, five are in CC view, and the other five are in MLO view.

#### 3.1.1. Mammography Database

The database that will be used for the analysis of different types of lesions will be mini-MIAS [21]. The types of abnormalities that this data set has are: (1) calcification (CALC), (2) well-defined masses (CIRC), (3) spiculated masses (SPIC), (4) other ill-defined masses (MISC), (5) Architectural Distortion (ARCH), (6) Asymmetry (ASYM), and (7) Normal (NORM). The database contains 322 images that have a resolution of 1024 × 1024. This database is quite small in the context of DL and VC, but it is widely used in the literature [22]. One hundred percent of the images will be divided into three parts: (a) training data set (80%), (b) validation data set (10%), and (c) the test data set with the remaining 10%. For each group, the data will be chosen carefully, leaving an example of each of the types of injuries contained in the dataset. The test data set will only be used to evaluate the proposed model, this implies that these data will never be used to train the model. DCNN architecture will be trained for the classification of lesions in breast images and the accuracy of the model will be analysed with different evaluation metrics [23]. This is described below.

#### 3.1.2. Deep CNN Architecture and Training

A Deep CNN architecture will be selected and evaluated: VGGNet-16 was chosen in this case study. This model was selected because it is a shallow architecture, compared to the most recent ones, and it does not require large computing capacity, since the availability of resources is limited for this project. To carry out the training, some modifications will be made to the original architecture of VGGNet-16, pre-trained on the ImageNet database. Among those modifications is the removal of the original classifier. Then a new classifier will be inserted, according to the selected database. Finally, the model will be trained.

#### 3.1.3. Pre-Training and Fine-Tuning

A promising alternative to training deep CNNs from scratch is transfer learning, which is essentially the use of pre-trained networks from different fields of application, usually carried out on natural images. Pre-trained models are being successfully applied in various tasks within the field of vision of computers (VC), which can act as a feature generator or as a basis for transfer learning [24]. In this way, there are two strategies applied to the transfer of learning that can be identified: (1) the use of pre-trained networks (pre-training) only as an extractor of characteristics and (2) making small adjustments (fine-tuning), on the weights of a pre-trained network, to achieve better performance on the data to be processed. Pre-training entails establishing pre-trained parameters rather than random ones while initialising networks. It is widely used in CNN-based models, since it has the benefits of accelerating learning and enhancing generalisation skills. Many approaches use this model trained on ImageNet 2012 as their reference deep model [25], and use fine-tuning for parameter tuning in accordance with the specific task that they have as their target, due to AlexNet’s good performance.

Table 2 shows the Results of comparing the study with some of the other studies that used datasets (mini-MIAS database). They were compared in terms of samples, methods, results, and the contribution of each study [26,27,28,29,30,31].

### 3.2. Tissue Classification Tool

Using convolutional neural networks (CNN), an automatic tissue classification CAD system was used. CNN’s are a type of artificial neural network (ANN) where neurons have receptive fields similar to those in the biological primary visual cortex. ANNs are an automatic processing tool inspired by the brain’s neural networks. This tool’s value is based on its ability to learn. Therefore, given a large enough set of input data, the network can learn a set of characteristics that allow it to classify new data. They are suitable for clinical decision-making due to their ability to learn complex data patterns not visible in the medical chart [32,33].

#### 3.2.1. Architectures and Layers of the CNN

**Figure 1:**
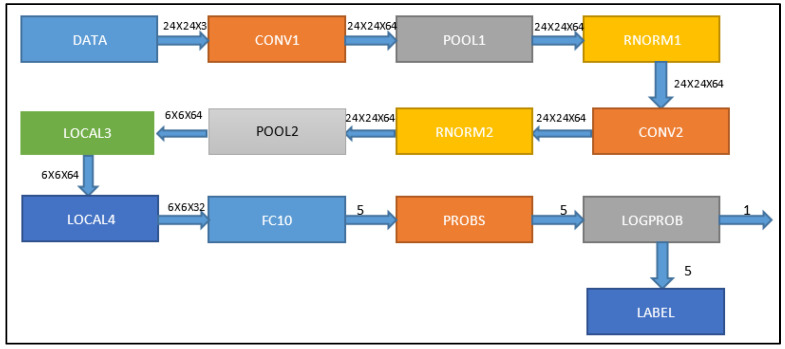
Structure of the convolutional neural network used in this work. Each of the layers is shown accompanied by the dimensionality of the input and output data.

The CNN used has a total of ten layers (Figure 1). In feature extraction, they are conv, pool, norm, and local. In the conv1 and conv2 convolutional layers, parts of the image are given convolutional operations. The pool1 and pool2 layers apply filters to reduce data in two of the three dimensions. However, the rnorm1 and rnorm2 layer neurons normalize the data, allowing similar characteristics to be detected in different images with different values. The final layers of this phase are local3 and local4, which operate similarly to convolutional layers but do not share weights. These layers are used in network classification. Layer fc10 neurons are fully connected and generate five outputs by multiplying the results of layer local4 by a weight matrix. In the first qualifying round, the probing layer neurons output five probabilities that the input image belongs to five classes. Finally, the log-probing outputs the mammogram’s most likely class. This layer also defines the network learning function “backpropagation,” which propagates output errors backward. The hidden layer receives a fraction of the overall error signal based on its contribution to the actual output. Each network layer receives an error signal describing its contribution to the total mistake. Then there is a phase called “adaptation,” in which the weights of the neurons are changed to cut down on the error. 

Layer Pool: By calculating an aggregate statistic from the surrounding outputs, the pooling layer substitutes for the network’s output at certain points. This aids in shrinking the representation’s spatial size, which lowers the amount of computation and weights needed. Each slice of the representation is subjected to the pooling procedure separately. A weighted average based on the distance from the center pixel is one of the pooling functions, along with the average of the rectangular neighbourhood and the L2 norm of the rectangular neighbourhood. However, max pooling is the most common method because it gives the most output from the neighbourhood.

##### Layer 2, Fully Connected

As in a conventional FCNN, all of the neurons in this layer are fully connected to all of the neurons in the layer before and after. Because of this, it can be calculated using a matrix multiplication followed by a bias effect, as usual. The representation between the input and the output is mapped using the FC layer. Layers of non-linearity after the convolutional layer, non-linearity layers are often added to make the activation map less linear, since convolution is a linear operation and pictures are anything but linear. Non-linear operations come in a variety of forms, the most common ones being:

##### Sigmoid

The mathematical formula for sigmoid nonlinearity is () = 1/(1 + e). It “squashes” a real-valued number into the range between 0 and 1. The gradient of a sigmoid becomes virtually zero when the activation is at either tail, which is a highly unfavourable sigmoid feature. Back propagation will essentially “kill” the gradient if the local gradient is too tiny. In addition, if the input to the neuron is only positive, the output of the sigmoid will either be only positive or only negative. This causes the weight gradient to change in a zigzag pattern.

##### Tanh

A real-valued number is condensed by Tanh to the range [−1, 1]. Similar to sigmoid neurons, the activation saturates, but unlike them, its output is zero-centered.

### 3.3. Methodology

**Figure 2:**
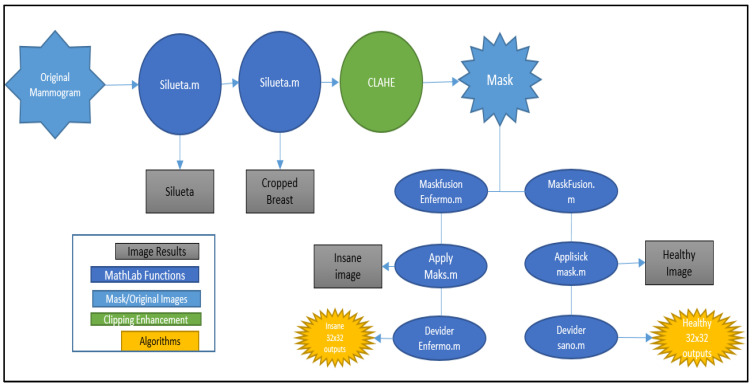
Diagram of the procedure for obtaining the cuts.

Figure 2 shows the steps that were taken to improve the contrast of the images and make them fit the classifier’s needs.

**Figure 3:**
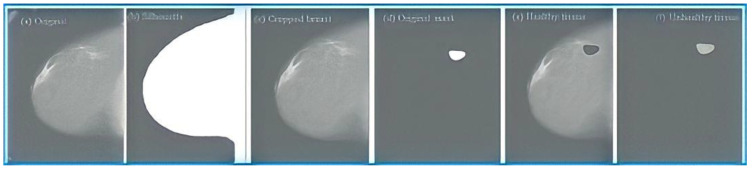
Images obtained during the training of the training set.

- First, the silhouette of the breast is obtained for each of the mammograms. This silhouette is obtained with equalization of the mammography histogram and a series of morphological operations that allow the contour of the breast to be obtained. Figure 3b shows that the result is a binary image that shows the silhouette of the breast.
- Secondly, the cropped breast is obtained by applying the silhouette obtained in the previous point to the original image, as shown in Figure 1. In the resulting image, the cropped breast will have the silhouette’s edges, but the breast tissue will remain unchanged. In this way, an image is obtained that contains only information about the breast, eliminating the label and other elements that could be in the background. Figure 3c corresponds to an image obtained in this phase.
- Next, the image enhancement phase takes place, where one of the two contrast enhancement algorithms is applied with the parameters detailed in Section 4. In this way, an enhanced image is obtained, characterised by an increase in contrast between the different tissues.
- After this, the enhanced images containing some pathology have a mask created by radiologists applied to them, which indicates the location of the anomaly (Figure 3d). This mask makes it possible to obtain, on the one hand, the abnormal tissue (Figure 3f), which corresponds to the interior of the mask, and, on the other hand, the healthy tissue, which corresponds to the rest of the tissue (Figure 3e).

For the neural network, healthy and unhealthy tissue images are divided into 32 × 32 fragments. The network requires that images be organized by class. Benign calcifications, malignant calcifications, malignant masses, and healthy tissue will be divided into five 32 × 32 images. The number of images in each subset varies, which may skew the results. To compensate for the neural network, we must introduce the same number of images into each type. The weighting is performed based on the folder with the fewest images, in this case, the 890 tissue sections associated with benign masses.

#### 3.3.1. HIW: Histogram-Based Intensity Windowing

The HIW method is an intensities-windowing extension. In IW, pixel intensities are determined by a linear transformation that maps the selected pixel set’s range of intensities to the device’s grey values. This allows a set of image intensities to span the device’s entire contrast range. Pixels outside the range are assigned the intensity value 0 (black) or 1 (white). The IW algorithm was tested on simulated masses introduced into dense breast tissue from digitized mammograms by the University of North Carolina’s Department of Biomedical Engineering [4]. They found that the windowing algorithm improved mass detection in mammograms, but that choosing the right parameters was critical, as they could significantly degrade the image.

##### Implementation of the Algorithm

The HIW algorithm divides the mammographic image into four sections: background, fatty breast tissue, dense breast tissue, and tumour masses and micro calcifications. The last two areas can be difficult to distinguish, especially when the breasts are dense due to their similar luminosity.

The window thresholds will be selected once the image has been segmented. The algorithm will then enhance the region or regions between the thresholds, mapping them to a range of intensities that contains all the available contrast, that is, between 0 and 1. After that, a similar image will be obtained, but with the region of interest enlarged.

The code In Matlab has four phases: image normalization, region labelling, region selection by lower and upper percentiles, and output image.

After normalization, the Matlab multi-thresh function is used to calculate a series of thresholds that label the image’s different tissues. To use this function, you must supply the mammographic image to be enhanced and the number of regions to be segmented. It returns an array of threshold values that divide the normalized image. For example, to segment an image into four regions, the output array contains the values of three thresholds. This data allows labelling image regions based on thresholds.

Then, choose the enhancing area. A higher value (value 1 of the normalized histogram) means we want to enhance pathological tissue, while a lower value means we want to enhance a different region (or regions). The second threshold should be set to a minimum and the maximum intensity value to a maximum. To obtain the enhanced image, the misadjust function has been used. This function takes as input parameters the normalized image, the lower and upper percentiles to be mapped, and the contrast values, which will always be 0 and 1.

### 3.4. Choice of Parameters

The parameters for HIW enhancement are the number of thresholds to segment the image and the lower and upper values to set as the function’s limit percentile.

**Figure 4:**
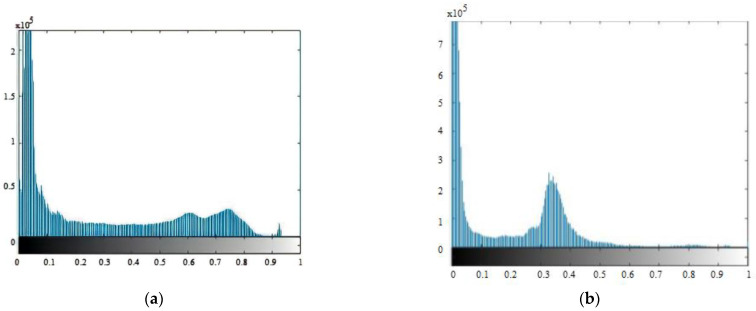
Histograms of mammograms in CC (**a**) and MLO (**b**) views.

**Figure 5:**
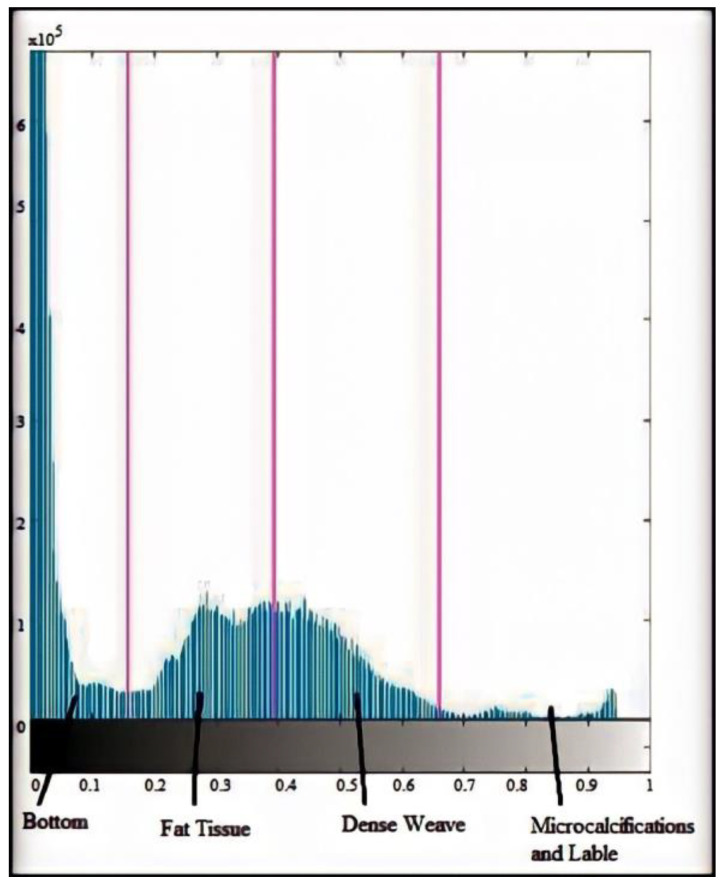
Thresholds on the histogram of a mammographic image in CC view and tissues that make up the image.

The histogram determines the number of regions. Figure 4 shows the normalized CC and MLO histograms. It corresponds to a dense breast, so the different regions of the breast are not easily distinguished. The MLO histogram shows a dense and fibrous breast. Four distinct regions can be seen here. The histogram flattens in the area of fibrous tissue of the breast; the second peak corresponds to the densest regions of the breast; finally, between intensities 0.6 and 1, a small number of pixels correspond to pathologies. In terms of the original image’s four regions: background, breast adipose (fat), dense tissue, and micro calcifications, Figure 5 depicts the histogram thresholds and tissues. Because masses, both malignant and benign, usually overlap or belong to the dense tissue region, the last label includes only micro calcifications. This is a major issue in dense-tissue mammography analysis.

### 3.5. CLAHE: Contrast Limited Adaptive Histogram Equalization

Contrast-limited adaptive histogram equalization (CLAHE) [34,35,36] is a technique based on adaptive histogram equalization (AHE), where the histogram of the resulting image is calculated from the histogram of a local window centered on each pixel, which is called the “neighborhood region.” It has been shown that the AHE algorithm can enhance areas of interest in medical images, although there is an over-enhancement of noise.

#### 3.5.1. Implementation of the Algorithm

The CLAHE algorithm consists of four stages: setting the input parameters, pre-processing, region processing, and interpolation.

- The image, the number of regions specified in rows and columns, the number of histogram bars used to establish the transfer function, and the normalised limit value between 0 and 1 must be established first.
- Secondly, the input data is pre-processed, whereby the real limit value is determined from the entered normalised value to adapt it to the characteristics of the image.
- A grey-level map results from processing each region. To do so, each region of the image is extracted, and a local histogram is created using the parameter bar count. The histogram is then fitted with the selected limit value and the region mapped.
- The final equalised image is obtained by interpolating the previous point’s results. This provides a final image from 4 nearby regions. Each extracted pixel is subjected to four transfer functions to obtain the output pixel, one for each region. After this, on each pixel, the final image is obtained.

For the implementation of CLAHE, the Matlab function has been used.

#### 3.5.2. Choice of Parameters

In the CLAHE algorithm, the following parameters can be modified, listed in Table 3.

**Figure 6:**
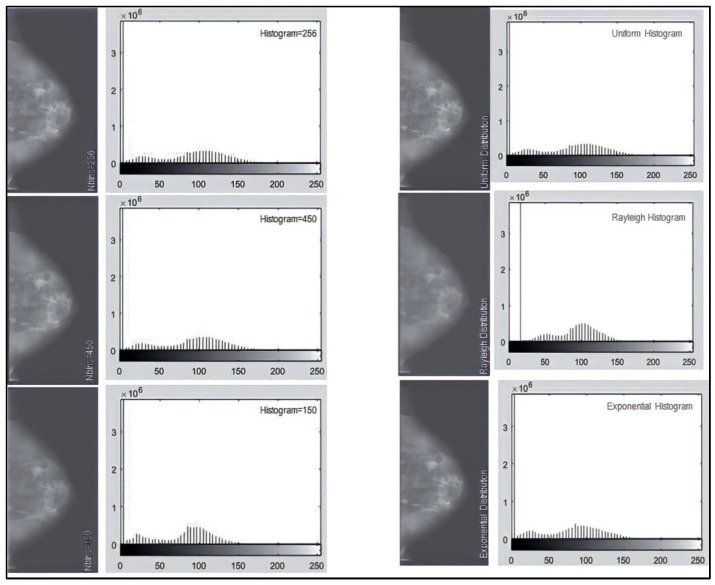
Distributions available in the CLAHE function and their histograms. From left to right uniform, Rayleigh, and exponential.

**Figure 7:**
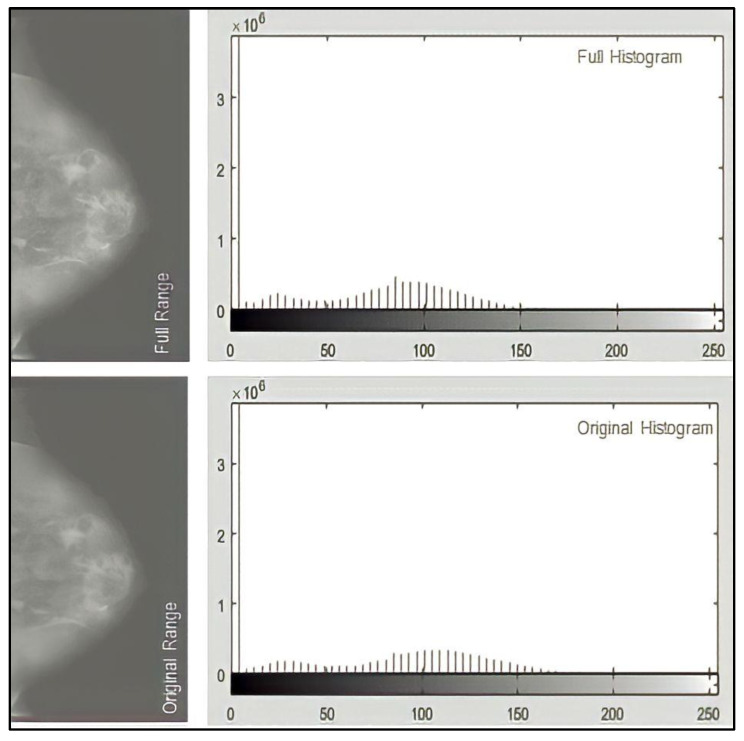
Ranges available in the CLAHE function and their histograms.

The best values for the parameters: the size of the regions, range, distribution, and alpha, are 256, complete, and uniform. When working with images as uint16 arrays, the range parameter is translated as [0, 255]. Alpha is not defined because it is not required for the full distribution. The results show that “256” is the best value for the number of equalized histogram bars. However, decreasing the number of histogram bars does not make a significant difference. It causes more pixels in the middle of the histogram, lowering the contrast between tissues. In Figure 6, the histograms of the three possible distributions are almost identical. The distribution is uniform throughout the work. Finally, the range parameter’s effects are examined. Figure 7 shows the images and histograms for its two possible values. Both histograms have very similar values. The range value will remain full throughout the job.

## 4. Results

### 4.1. Description and Organization of Tests

To determine which parameters produced the best results and if the improvement algorithms increased the number of hits in the categorization of mammograms, several experiments were conducted. While the difference between the various breast tissues is increased by the many tests of both algorithms, some are more pronounced than others. The experiments that were run are listed in Table 4, along with the method and input parameters.

The results are presented for each test as a table, which includes specific information about the classification accuracy attained for each set of images. By definition, precision is:

(1) $$\mathrm{Precision}=\frac{TP+TN}{TP+TN+FP+FN}$$

The terms *TP* and *TN* stand for the total number of positives and negatives, whereas *FP* and *FN* stand for the terms false positives and false negatives, respectively. *TP* and *TN* also stand for the total number of genuine positives and true negatives. The ratio of true positives to the whole set of all components is that. Additionally, a confusion matrix is used to present each test’s findings, allowing users to see them as a color-coded chart. Each row represents each instance in the real class, whereas each column shows the number of predictions for each class. It makes it possible to practically collect data on the network’s accuracy as well as data on the false positives and false negatives discovered throughout each test.

### 4.2. Performance Metrics and Evidence

The performance of two histogram-based contrast enhancement algorithms, HIW and CLAHE, for an automatic classification CAD system was measured using accuracy, precision, recall, f1-score, and support.

Test-0: To begin with, a preliminary test using unprocessed images was conducted. To compare the outcomes of the other tests, this test is used as a benchmark.

The findings of test-0, which had an accuracy of 75%, are shown in Table 5. With the exception of the set of healthy photos, when the accuracy falls to 56%, this accuracy is more than 75% in all groups of photographs.

**Test-1:** The HIW algorithm was utilized in the first test. The picture is divided into five categories of tissue using four thresholds: fundus, fatty tissue, dense tissue, masses, and microcalcifications. The smallest value is the second threshold, which represents thick breast tissue, and the highest value is intensity 1. The image’s densest structures, diseased structures, and microcalcifications are all increased in this manner.

The findings of test-1, which had an accuracy of 67%, are shown in Table 5. The analysis of the picture sets reveals that the proportion of benign masses and malignant calcifications exceed 75%, while the proportion of benign masses and malignant calcifications is 62%. Last but not least, just as in the reference test, the healthy picture set has the lowest accuracy (59%), making it the only one that improves upon the reference as it drops in the others. It should be noted that the overall accuracy (67% vs. 75%) is lower than the reference test.

Test-2: The HIW algorithm was once again utilized in the second test, but this time, the three thresholds in the picture were separated into four tissues: the fundus, fatty tissue, dense tissue (which may contain potential masses), and microcalcifications. The second threshold sets the lower limit for the growth of tissue pathology and density. The diseased structures show out more as a result of this threshold selection, which prevents the fibrous tissue from shining out.

Table 5 displays the test-2 results, which showed a 64% accuracy rate. When analyzing each group of photos separately, it should be observed that the accuracy for malignant calcifications is 80%, compared to 61% and 64% for the sets of benign masses and can-cerous masses, respectively. The accuracy of the healthy picture set and benign calcification are only 59% and 54%, respectively. Each pair of photos has lower overall and per-image precisions than the reference found in test-0.

Test-3: The third test, the last one in which the HIW algorithm is used, recalculates three image thresholds, but the first threshold is set as the lower limit to boost fatty tissue along with denser and diseased tissue. In other words, the data about low-intensity structures, such as the triangle’s edge, displays the outcomes of test-3, which yielded a 74% accuracy. Only the images in the “malignant calcifications” folder are consistently correct more than 80% of the time. The accuracy of the healthy photos is 59%, whereas it is 74%, 79%, and 75% for the sets of benign calcifications, benign masses, and malignant masses, respectively. In this instance, despite having poor accuracy, only the group of healthy photos outperforms the reference (59% vs. 56%), even if the remaining precisions are the same or just a little less precise.

**Figure 8:**
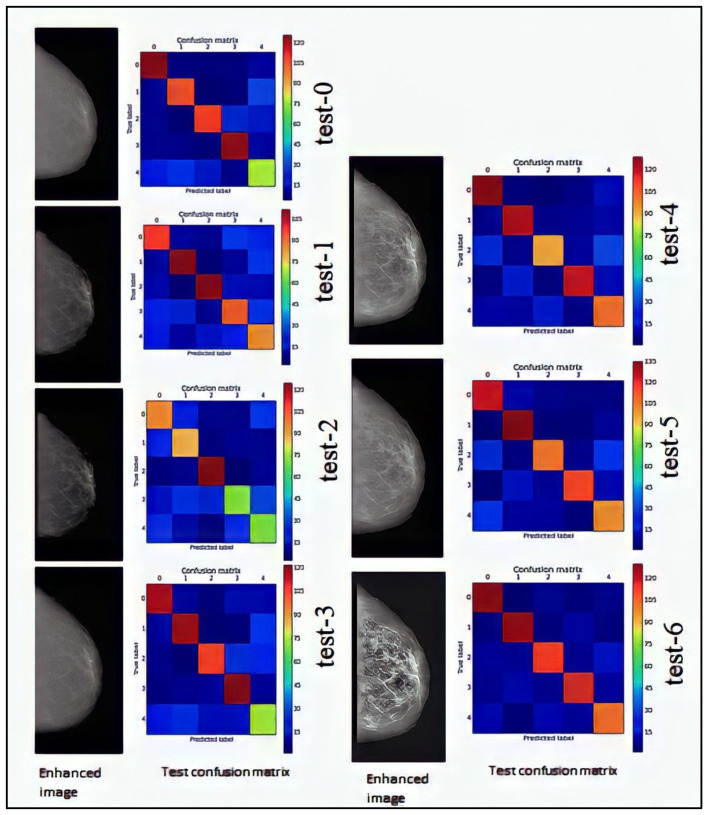
Results obtained during the test-0 to test-6.

**Test-4:** This test made use of the CLAHE algorithm. In this instance, the limit value is set to 0.01 and the size of the tiny sections employed by the method is fixed in 8 × 8 matrices. The difference between the denser structures and the diseased and fibrous structures becomes more pronounced, as shown in Figure 8.

The findings of test-4, which produced an accuracy of 78%, are shown in Table 5. The accuracy of each set as a whole is better than 80%, with the exception of the healthy photos, whose accuracy is 63%. Only the malignant calcifications group has a worse accuracy than the reference.

**Test-5:** The CLAHE algorithm was used in this test. The areas’ sizes are changed to 3 × 3 matrices, but the limit value, which remained at 0.01 from the previous test, stays unchanged. Figure 8 shows how all the different tissue types have been highlighted, which heightens the contrast between the densest tissue and the others.

The findings of test-5, which had an accuracy of 80%, are shown in Table 5. Individual accuracy scores for benign masses, malignant calcifications, and malignant masses are 80, 82, and 89%, respectively, while scores for benign calcifications and healthy pictures are 79 and 72%, respectively. Only the group of malignant calcifications in this instance is a little bit under the reference value (83%).

**Test-6:** The CLAHE method was utilized in the final test, with the area size set to 8 × 8 matrices and the limit value set to 0.05. Figure 8 illustrates how several breast tissues seem to be much increased. They may even seem too heightened to the unaided eye, heightening the contrast between various areas of the same tissue. On the other hand, it is clear how drastically different the denser tissues are from the rest of the structures.

The findings of test-6, which had an accuracy of 81%, are shown in Table 5. Only healthy photos have an accuracy of 76%, which is less than 80%. All the others are more accurate than 81%. Although the overall accuracy is better than the reference, the precision for the particular malignant groups—masses and calcifications—is worse.

## 5. Discussion

The study aimed to analyze the differences in the detection of simulated masses by enhancing the images with HIW and CLAHE to find the algorithm that obtained the best results. This analysis was based solely on expert radiologists’ and students’ visual detection of the masses. They concluded that these are related as follows:

It is important to highlight the importance of the choice of parameters, since only one set of parameters obtained better results than the original images. In the case of the HIW algorithm, the best results were obtained by setting the minimum percentile at 20%, while in the case of CLAHE, a combination of parameters capable of surpassing the images without a preprocessor was not found, but it was equal. The tests carried out in this TFG do not agree with those offered by said study.

Table 6 shows the precision data and errors obtained in each test. In test-0, considered the reference test because it was performed with the original images, an accuracy of 75% was obtained. Based on this information, the other tests are considered positive if the total precision result is higher than the reference, and negative if it is lower.

The first test’s accuracy is 67%. It is a negative test since the system’s automated categorization is reduced rather than improved by the HIW algorithm when these settings are used. Figure 8 demonstrates how boosting diseased and thick tissues creates pictures with low-contrast masses and high-contrast micro calcifications. Despite this, the generated photos lack contrast between the breast and backdrop, and have poor intensity. This may account for the reduced overall accuracy.

With a total accuracy of 64%, the second test similarly yields a negative result. The diseased tissue of high intensity and the dense tissue of comparable intensity, both of which lie between the third and fourth thresholds, are enhanced using three rather than four thresholds in this test. The maximum intensity is set higher, while the second threshold is lower. Pathologies, thick tissues, and fibrous or fatty tissues all benefit from this. The difference between tissues is less in this instance, as shown in Figure 8. In comparison to the prior and reference tests, this test has lower individual precisions of benign and malignant tumours. This indicates minimal contrast between components over the whole breast.

The third test, like the first two, shows a somewhat lower overall precision than the reference: 74% vs 75%. Figure 8 depicts an improved mammogram. The initial threshold is then reset, this time with a maximum value. This results in the enhancement of only low-r intensity structures.

In the fourth test, the CLAHE algorithm achieved 78% accuracy. Figure 8 demonstrates how the individual accuracy of benign and malignant tumours rises as tissue density increases. Micro calcifications, on the other hand, are substantially amplified, with intensities near to one. Self-classification becomes more challenging due to increasing dense tissue structures. Given the findings, it’s probable that the algorithm is considering more nearby pixels than are required to obtain a satisfactory enhancement and contrast increase.

It is 80% accurate to use the fifth test. The regions are now 3 × 3, but the limit factor is the same. Denser structures and the remainder of the breast tissue are more contrasted in Figure 8 than they are in Figure 7. The margins of the breast are amplified despite this, bringing them closer to the intensities of the breast tissue, and perhaps preventing network categorization. Because of the contrast between them and the nearby tissues, microcalcifications maintain their high intensity.

The sixth test, which has the greatest overall score of the tests, has a score of 81%. With the same 8 × 8 sections and 0.05 intensity limits, the CLAHE algorithm is applied once again. As shown in Figure 8, there is the greatest difference between the resx8 sections with a 0.05 intensity limit and the densest structures, whose intensity declines to almost zero. The contrast between the densest structures, whose intensity drops to almost nil, and the remainder grow, as seen in Figure 8. Malignant and benign mass individual precision increases, while calcification precision decreases. This may be the case because the tissues with high intensities shown in these pictures are microcalcifications and masses. The neural network is assisted in learning the various tissue types and their categorization as healthy or diseased by the reduction in the intensity of non-dense tissues.

The greatest accuracy for benign calcifications in test-6 was 81%. The optimum algorithm in this situation is CLAHE, which has 8 × 8 areas and a 0.05 limit. Notably, HIW algorithm accuracies are up to 10% lower than CLAHE algorithm accuracies, which all surpass the reference. This demonstrates that the CLAHE algorithm performs better than the HIW method in classifying benign calcifications.

The reference value for benign masses is 79% accurate. Only the third test meets or surpasses the reference when using the HIW algorithm. In all three tests, the CLAHE algorithm’s accuracy is greater, with test-6 having the greatest accuracy at 85%.

One test does not outperform the reference precision for malignant calcifications among the six. All of the scores are in the 80% range; however, only test-5’s 82% score comes close to the 83% reference. Therefore, it is recommended to avoid using any algorithm while analysing mammograms for malignant calcifications. In the three studies, the HIW algorithms’ accuracy for malignant tumours was up to 20% lower. The results of the CLAHE algorithm tests all surpass this precision, with the fifth reaching 89%, except test six, which serves as the reference. As a result, the CLAHE algorithm increases accuracy, particularly when the test-5 test parameters are used. Finally, the accuracy of the healthy pictures is substantially lower than that of the other groups. Despite this, all but one of the tests surpassed the benchmark of 56%. This is significantly more accurate than the 76% of the CLAHE algorithm. The test with the highest degree of accuracy for each set is shown in Table 7, along with the method and input parameters.

Test 6 yields the greatest results in three of the five picture sets, according to Table 7. Therefore, for an automated classification CAD system, the CLAHE algorithm with an 8 × 8 area size and 0.05 limit values produces the best results. The most accurate groups would be those of benign calcifications, benign lumps, and healthy tissue. Although the sets of malignant masses and malignant calcifications were not pre-processed using the superior method, there is little difference between them, since both have individual precisions that are more than 80%. Additionally, test-6 produced the highest global accuracy, confirming the usage of the algorithm throughout the pre-processing stage of the CAD system. As a consequence, all tests would have a tool with an overall accuracy of 81% and individual precisions of around 80%.

Table 8 shows the results obtained. The precision achieved by the proposed model was 62%.

In our study, a DL model was presented that implements DCNN architecture to classify different types of lesions directly from breast ultrasound scans. It showed that it is difficult to achieve high precision when there is not a database with a large number of examples for each type of lesion. However, it was possible to observe how the different training strategies influence to counteract this situation. Future work could include exploring other architectures and testing this model with larger datasets or using multiple data sources.

## 6. Conclusions and Future Studies

Comparing the results obtained with the two algorithms, it can be seen how the CLAHE algorithm obtains a higher precision, exceeding the reference precision. This may be because the HIW algorithm, in addition to not taking into account the noise present in the image, only enhances certain types of tissues, while CLAHE enhances the entire image, increasing the contrast between the different structures. On the other hand, it should be noted that not all image subsets are equally affected by the algorithms. The best result obtained for malignant micro calcifications is with the image without enhancement, while for benign masses, malignant masses, benign micro calcifications, and healthy tissue, the precision is higher by enhancing them with the CLAHE algorithm. This demonstrates that depending on the type of pathology to be detected, it is more convenient to use the CLAHE algorithm or not.

Given the results obtained throughout the work, it can be stated that the preprocessing of mammographic images using histogram-based contrast enhancement algorithms can provide numerous improvements in the preprocessing stage of a CAD system using neural networks. Convolutional, giving rise to a tool capable of detecting and classifying abnormalities in breast tissue, can also be used. On the other hand, the influence of different factors has been demonstrated, not only in the algorithms but also in the entire process, until the result of the CAD system is obtained. Despite this, it is necessary to continue studying these factors to find the best possible combination of them to increase the accuracy of the network.

To conclude, we want to highlight that the results obtained in this TFG open a new path of study that allows us to improve the CAD system, facilitating the detection and classification of cancer. In this way, the system could constitute a fundamental tool to help physicians, which helps them in decision-making. Thus, it is possible to increase the possibility of treating cancer in its initial stages, improving the quality of life of patients who suffer from it, and, ultimately, reducing the mortality caused by this disease.

### 6.1. Future Lines

It would also be interesting to combine contrast enhancement algorithms with others such as edge detection. In this way, it would be possible to delimit all the structures present in the breast, including the pathological tissue, introducing new information to the neural network that could facilitate its learning and give rise to better precision in the classification.

### 6.2. Limitations

This study compared the performance of two histogram-based contrast enhancement algorithms, HIW and CLAHE, for an automatic classification CAD system. Comparing the results of the two algorithms reveals that the CLAHE algorithm achieves higher precision than the reference precision. In addition to not accounting for image noise, the HIW algorithm only enhances specific tissues, whereas CLAHE enhances the entire image, increasing contrast between various structures. Using histogram-based contrast enhancement algorithms to preprocess mammographic images can significantly improve the preprocessing stage of a CAD system using neural networks. Convolutional neural networks were used to detect and classify breast tissue abnormalities. In addition, the images used in this work come from the DDSM database.

### 6.3. Future Work

Our framework is flexible and can be readily utilized for other diseases, other forms of risk modeling, and other definitions of early detection benefits or screening costs. We expect the utility of history-based techniques to increase. As a result, by using histogram-based techniques, we may be able to better treat cancer in its early stages, improve cancer patients’ qualities of life, and ultimately reduce cancer mortality. The mammogram is the best tool for breast cancer screening at the moment, with about 85% accuracy. Moreover, in the future, I will use performance metrics such as sensitivity, specificity, precision, F1-score, Matthew correlation coefficient, and ROC curves.

## Figures and Tables

**Table 1:** Critical literature review.

No.	Article Cite	Sample and Methodology		Results	Contribution of Study
1	Ragab et al. (2019) [7].	1-The digital mammogram database (MIAS) 2-The Digital Mammography Dream Challenge	1-Computer-aided detection (CAD) system is introduced. 2-K-nearest neighbor (k-NN) and decision tree classifiers were used to classify the normal and abnormal lesions. 3-Multiple classifier systems (MCS) were constructed as it usually improves the classification results.	The results showed that the proposed CAD system could correctly classify mammogram samples into normal and abnormal lesions.	The contribution of this study is the use of mammography images, a novel CAD approach, given to categorize benign and abnormal mass lesions. The research focuses on creating a CAD system by merging two widely used datasets.
2	N. Kharel et al. (2017) [8]	Use CLAHE and Morphology method to enhance image for further computation in CAD system for early diagnosis of breast cancer using mammogram images.		According to the study, the intended solution can improve mammogram images for segmentation, feature extraction, and classification during subsequent processing.	The study contributed to improving the use of a hybrid approach for the early detection of breast cancer utilizing mammography images.
3	S. F. Mat Radzi et al. (2020) [9]	Thirty mammogram images from The Cancer Imaging Archive (TCIA) datasets were randomly selected as subjects.	1-Use the Contrast Limited Adaptive Histogram Equalization (CLAHE) 2-Adaptive Histogram Equalization (AHE) techniques on a benign tumor of two-dimensional (2D) mammography images.	Since the previous two strategies produced repeatable descriptors, semiautomatic segmentation with image enhancement using the CLAHE algorithm produced the best results and was a superior option to human delineation.	This study’s contribution is to assess the reproducibility of radiomics features obtained from manual delineation and semiautomatic segmentation following enhancement using Contrast Limited Adaptive Histogram Equalization (CLAHE) and Adaptive Histogram Equalization (AHE) techniques on a benign tumor of two-dimensional (2D) mammography images.
4	A. Vidyarthi et al. (2019) [10]	The proposed architecture is tested using the BreakHis dataset, which consists of 7909 total images, 2480 of which belong to the benign class and the remainder to the malignant class.	1-Use the traditional CNN architecture to classify the images into benign and malignant 2-Contrast Limited Adaptive Histogram Equalization (CLAHE) followed by the watershed algorithm.	According to the experimental findings, the proposed hybrid architecture performs better than conventional techniques in terms of accuracy, with gains of about 3%.	The study contributed to the present hybrid CLAHE-deep convolutional network architecture for classifying breast microscopic images.
5	Chaudhury S et al. (2022) [11]	Mammograms in the MIAS database	1-Use the CLAHE algorithm. 2-Use the -means algorithm 3-The images are then classified using techniques such as fuzzy SVM, Bayesian classifier, and random forest to determine their classification.	The MIAS database, which is available online, contains 322 mammography pictures of the right and left breasts. There were 322 total photographs, of which 207 were determined to be normal and 64 to be benign. Of these, 51 were determined to be malignant. The remaining 72 photos were utilized to evaluate categorization approaches after 250 images were used to train machine learning algorithms.	In this paper, a machine learning and image processing framework for breast cancer is described. This framework uses a collection of mammography images as its input data set. The quality of these photographs is then improved by image processing utilizing the CLAHE approach. CLAHE.
6	Bacha S et al. (2022) [12]	1-The Mammographic Image Analysis Society (MIAS) 2-The Wisconsin breast cancer database (WBCD)	Diagnosis of breast cancer disease based on an evolutionary algorithm known as Differential Evolution (DE) of a Radial-Based Function Kernel Extreme Learning Machines (RBF-KELM).	In comparison to traditional methods, the outcomes were good.	In the work that is being described is a differential evolution method, termed DE-RBF-KELM, and the RBF-KELM classifiers are used to construct an expert diagnosis system for breast cancer illness. The recommended DE-RBF-KELM method’s competitiveness is evaluated using static techniques including classification accuracy, F-score, and sensitivity and specificity analysis. Additionally, the effectiveness of the recommended design is contrasted with more sophisticated methods.
7	Suh YJ et al. (2020) [13]	The performance was tested using 301 combined photos from 284 participants, and the results were contrasted with a meta-analysis of 12 earlier deep learning experiments.	Our deep learning models would help to interpret digital mammography to identify patients with breast cancer. Using this strategy, the burden on radiologists could be reduced considerably.	Using digital mammograms that were initially gathered from our institution, we created two CNN models for automated breast cancer diagnosis in the current research. The DenseNet-169 and EfficientNet-B5 models were trained using a concatenation of two pictures per breast. With DenseNet-169 and EfficientNet-B5, the mean AUC was 0.952, 0.005, and 0.954 0.020, respectively. In sub-groups containing breasts with decreased parenchyma density, the mean AUC rose.	The study contributed to develop and validate a deep learning model that can automatically identify malignant breast lesions in Asian women who undergo digital mammography, and research the model’s performance in relation to the grade of breast density.
8	H. -C. Lu et al. (2019) [14]	In this study, we preprocessed over 9000 mammograms using a median filter, contrast-limited adaptive histogram equalization, and data augmentation. We then trained a classified model using a convolutional neural network.		The results of the experiment showed that the model with preprocessed images significantly outperformed the model without preprocessed images in terms of accuracy.	This research used traditional X-ray imaging as the basis for its breast cancer screening technique. The sensitivity and specificity of the diagnosis were primarily dependent on the radiologist’s expertise, and questionable diagnoses are common due to time constraints on resolution and worries about litigation brought about by incorrect diagnoses or missed lesions.
9	Escorcia-Gutierrez J et al. (2022) [15]	Using a benchmark dataset, the results are analyzed under several performance measures	1-Use Deep Convolutional Neural Network based Residual Network (ResNet 34) 2-The wavelet neural network (WNN) for classification of digital mammograms for the detection of breast cancer.	The simulation results showed that the ADL-BCD model outperformed the most recent techniques in terms of various metrics.	This study uses digital mammograms to demonstrate an automated deep learning-based breast cancer diagnostic.
10	Singla C et al. (2020) [16]	The Mammographic Image Analysis Society has put the suggested methods into practice. It has 322, which is a large number of images.	The segmentation technique used is thresholding, which is applied to the enhanced image.	The categorization of breast lesions is a method that may be used to limit the death rate from breast cancer. With its aid, we can significantly lower this rate. Mammogram images are essential for the diagnosis of breast cancer.	The study contribution: used the segmentation approach to check if the categorization of breast lesions, which may be either benign or cancerous, was performed properly. It is possible to significantly lower this rate with the aid of this technique.
11	J. Dabass et al. (2019) [17]	Using MIAS database	Histogram equalization (CLAHE) and entropy-based intuitionistic fuzzy method.	Results from experiments show that the suggested method provides higher visual quality. In comparison to a number of state-of-the-art algorithms, it offers high values of subjective and quantitative measurements.	In this paper, the contrast of digital mammography pictures is projected to be improved using contrast limited adaptive histogram equalization (CLAHE) and entropy-based intuitionistic fuzzy techniques.
12	Ragab et al. (2021) [18].	1-The digital database for screening mammography (CBISDDSM). 2-The mammographic image analysis society digital mammogram database (MIAS).	Using Computer-aided diagnosis (CAD) system based on feature extraction and classification using DL techniques.	When compared to cutting-edge CAD systems, the accuracy obtained utilizing deep features fusion for both datasets was shown to be the greatest. In contrast, accuracy did not increase when PCA was applied to feature fusion sets, but the computational cost increased as execution time shrank.	In this study, a novel CAD system is proposed to investigate the fusion of different features extracted from various DCNNs in order to select the best combination of features, which increases the CAD system’s accuracy.

**Table 2:** Comparison between the results achieved and the results of previous related studies based on the same dataset.

Article Cite	Sample and Methodology		Results	Contribution of Study
Kavitha et al. (2022) [26]	Mini-MIAS dataset and DDSM dataset.	Thresholding-based Segmentation with DL enabled Capsule Network (OMLTS-DLCN) breast cancer diagnosis model utilizing digital mammograms.	Using the benchmark Mini-MIAS dataset and DDSM dataset, the diagnostic results of the proposed OMLTS-DLCN approach are explored. The experimental results demonstrate the OMLTS-DLCN model’s improved performance, with accuracy values of 98.50 and 97.55% on the Mini-MIAS dataset and DDSM dataset, respectively.	The study examined the use of computer vision and deep learning (DL) techniques for performing breast cancer diagnosis.
Li Y. et al. (2013) [27]	Used 322 digitized mammograms from the Mini-MIAS database and 100 mammograms from the DDSM database.	Segmentation the pectoral muscle in mammogram.	The results show that the suggested method can be utilized successfully as a preprocessing step for additional mammography analysis, with acceptable rates of 90.06% and 92% for the mini-MIAS database and the DDSM database, respectively.	The segmentation of the pectoral muscles using an efficient method is suggested in this research. This technique makes use of the pectoral muscle’s homogeneous texture and high intensity deviation, which are both significant anatomical characteristics.
Sheshadri. Et al (2006) [28]	The data base (mini-MIAS database).	Classify the breast tissue based on the intensity level of histogram of a mammogram.	The outcomes of the trials clearly demonstrate the emergence of any defect in breast tissue growth. This technique was developed to reduce the amount of time and computing complexity required to analyze any given mammogram, and it has achieved accuracy levels of up to 78%. This is a fundamental stage in creating a CAD for mammo-analysis.	The contribution of this study is to classify breast tissue according to the intensity level of a mammography’s histogram. Statistical characteristics of a mammogram are extracted using straightforward image processing methods. The suggested method makes use of texture models to reproduce the breast’s mammographic appearance.
Zebari DA, Et al (2021) [29]	The data base (mini-MIAS database).	Hybrid thresholding and the machine learning method are used to derive the region of interest (ROI).	The results show that the proposed method yields better results on the INbreast dataset in the single-dataset evaluation, whilst better results are obtained on the remaining datasets in the double-dataset evaluation. The proposed approach outperforms other state-of-the-art models on the Mini-MIAS dataset.	The contribution of the study is the classification of benign or malignant breast cancer from mammogram images using Hybrid thresholding and the machine learning method to derive the region of interest (ROI).
S. Charan. et al. (2018) [30]	The data base (mini-MIAS database).	The methods that were used in this study were Machine learning and deep learning, or neural networks, which is one of the techniques that can be used for the classification of normal and abnormal breast detection.	Experimental results have been obtained which depict the efficacy of deep learning for breast cancer detection in mammogram images and encourage the use of contemporary deep learning-based feature extraction and classification techniques in a variety of medical imaging applications, particularly for the detection of breast cancer.	The contribution of the study is the detection of breast cancer using machine learning, which can help medical professionals to diagnose the disease with more accuracy, and deep learning, or neural networks, are one of the techniques which can be used for the classification of normal and abnormal breast detection.
Vishrutha V. et al. (2014) [31]	The data base (mini-MIAS database).	The method consists of three steps: The first step is to find region of interest (ROI). The second step is wavelet and texture feature extraction of ROI. The third step is classification of detected abnormality as benign or malignant using support vector machine (SVM) classifier.	The proposed method has achieved 92% accuracy.	The contribution of study is detection breast cancer microcalcifications and circumscribed masses, and also classifies them as benign or malignant. The proposed method consists of three steps: The first step is to find region of interest (ROI). The second step is wavelet and texture feature extraction of ROI. The third step is classification of detected abnormality as benign or malignant using support vector machine (SVM) classifier.

**Table 3:** Modifiable parameters in the algorithm.

Parameter	Value
Size of the regions	The parameter that sets the dimensions of the regions. It is an array of dimensions [M, N] where M specifies the number of rows and N the number of columns of the regions where the contrast transformation will be applied.
limit value	Real scalar in the range [0 1] defines the limit value.
Number of histogram bars	A positive integer scalar that specifies the number of bins in the histogram. The higher the number, the greater the dynamic range.
Range	Sets the output range of the image. It can take original value, where the output range is limited to the original image, or full, where the range depends on the type of image used.
Distribution	Specifies the distribution of the histogram that is used to perform the transformation. It can be of three types: uniform, where the histogram is uniform or flat; Rayleigh, bell-shaped; or exponential with exponential or curved histograms.
Alfa	A positive real scalar that specifies the distribution parameter.

**Table 4:** Description of the tests carried out.

Test Name	Algorithm	Parameters
test 0	No preprocessors	
Test 1	HOW	Number of thresholds: 4
		Lower threshold: 2
test 2	HOW	Number of thresholds: 3
		Lower threshold: 2
test 3	HOW	Number of thresholds: 3
		Lower threshold: 1
test 4	CLOSE	Region Size: [8 8]
		Limit value: 0.01
test 5	CLOSE	Region Size: [3 3]
		Limit value: 0.01
test 6	CLOSE	Region Size: [8 8]
		Limit value: 0.05

**Table 5:** Results obtained during the tests. The total precision is displayed along with the individual precisions for each set of images separately.

	Precision (%)						
Set of Images	Test-0	Test-1	Test-2	Test-3	Test-4	Test-5	Test-6
Benign calcifications	0.75	0.62	0.59	0.74	0.81	0.79	0.82
Benign masses	0.79	0.76	0.61	0.79	0.81	0.8	0.85
Malignant calcifications	0.83	0.78	0.8	0.81	0.8	0.82	0.8
Malignant masses	0.82	0.62	0.64	0.75	0.84	0.89	0.81
Healthy tissue	0.56	0.59	0.54	0.59	0.63	0.72	0.76
Total	0.75	0.67	0.64	0.74	0.78	0.8	0.81
Error	0.25	0.32	0.36	0.26	0.22	0.19	0.18

**Table 6:** Accuracy and error obtained in each of the tests.

Test	Precision (%)
Test-0	75
Test-1	67
Test-2	64
Test-3	74
Test-4	78
Test-5	80
Test-6	81

**Table 7:** Test that offers the best precision for each set of images, indicating the algorithm and its parameters.

Set of Images	Best Result	Parameters
benign calcifications	Test-6: CLAHE	[8 8] 0.05
benign masses	Test-6: CLAHE	[8 8] 0.05
benign calcifications	Test-0: reference	
malignant masses	Test-5: CLAHE	[3 3] 0.01
healthy tissue	Test-6: CLAHE	[8 8] 0.05

**Table 8:** Results.

Type of Lesion	Precision	Recall	f1-Score	Support
ARCH	0	0	0	5.25
ASYM	0	0	0	3.15
CALC	0.34	0.79	0.46	8.4
CIRC	0.21	0.35	0.26	6.3
MISC	0.15	0.26	0.19	4.2
NORM	0.9	0.5	0.65	54.6
SPIC	0.53	0.42	0.46	5.25
Average/total	0.65	0.45	0.5	0.86

## Data Availability

Not applicable.
